# Natural and Strategic Generosity as Signals of Trustworthiness

**DOI:** 10.1371/journal.pone.0097533

**Published:** 2014-05-15

**Authors:** Diego Gambetta, Wojtek Przepiorka

**Affiliations:** 1 European University Institute, Department of Political and Social Sciences, San Domenico di Fiesole (FI), Italy; 2 Nuffield College, Oxford, United Kingdom; 3 University of Oxford, Department of Sociology, Oxford, United Kingdom; Universidad Carlos III de Madrid, Spain

## Abstract

We exploit the fact that generosity and trustworthiness are highly correlated and the former can thus be a sign of the latter. Subjects decide between a generous and a mean split in a dictator game. Some of them are informed from the start that afterwards they will participate in a trust game and that their choice in the dictator game may matter; others are not informed in advance. In the trust game, before trusters decide whether or not to trust, some trustees can reveal (or conceal) only their true choice in the dictator game, while others can say to trusters, truthfully or otherwise, what they chose. We find that a generous choice made naturally by uninformed trustees and reliably revealed is more effective in persuading trusters to trust than a generous choice that could be strategic or a lie. Moreover, we find that, when they can, mean subjects lie and go on to be untrustworthy.

## Introduction

Since its Spencian inception signalling theory has focused on the production of signals and on its costs [1]. In its conceptual framework, signallers embark on certain activities aimed at persuading receivers of the truth of some state of affairs, often of some unobservable quality of theirs, such as their physical fitness or trustworthiness. How signals, once produced, are then displayed to receivers is implicitly assumed as unproblematic. If the receiver can observe someone running a marathon, for instance, the receiver can infer that this person has energy and stamina to a degree which could not be mimicked; here the production of the signal and its display are achieved by the same action. However, *production* and *display* are often distinct operations. We produce a signal, but then display only indirect evidence of it. The employer observes our *certificate* as proof of our schooling, and not our having been to school; our partners in crime observe our *scars* as evidence that we can both fight and survive; we can infer a person's energy by watching a *video recording* of the marathon rather than the run itself. Moreover, the evidence of the same signal can at times be re-displayed with efficiency gains—we do not go back to school every time we apply for a job [2].

Empirically and conceptually it makes perfect sense to distinguish between signal production and display, but the ability to produce the evidence of one's past actions is not coextensive with the ability to produce the actions themselves. One unable to get a degree can still succeed in forging a degree certificate. Why then do myriads of employers hire job applicants based on the degree certificates these applicants show them? Here we argue that type separation at the level of display can work as well as it works at the level of production. Mimics, who did not carry out the action, should find it too costly (relative to the benefits) to manufacture the evidence that they did, while producing the evidence should be affordable by the actor who truly did carry out the action. In other words, the occurrence of a past action which signals the presence of an unobservable property of the actor becomes itself a new, not directly observable property, and the evidence that it occurred becomes a new, second order signal.

Separating signal production from signal display instigates a further claim, which is central to our paper. Actions, which can subsequently be used as signals, are not always carried out anticipating their future information value; people are not strategic all the time in all they do, and often produce the “raw material” of potential signals with other reasons in mind. The interesting aspect of actions which are carried out, as it were, ‘naturally’ is that the production costs do not need to be type separating to be reliably informative. The simplest of gestures, which in strategic situations could be easily mimicked and thus distrusted, can become valuable. When agents do not anticipate the signalling value of their actions, those who behave kindly, for instance, do so because they are kind-hearted, while those who are not kind-hearted do not behave kindly because they do not expect any reward from doing so. Natural actions can thus make reliable communication more efficient, because cheaper for the honest signaller; this is so provided that the type separating conditions apply to the displayable evidence that a given action has both occurred *and* that the actor did not anticipate its future information value.

**Figure 1:**
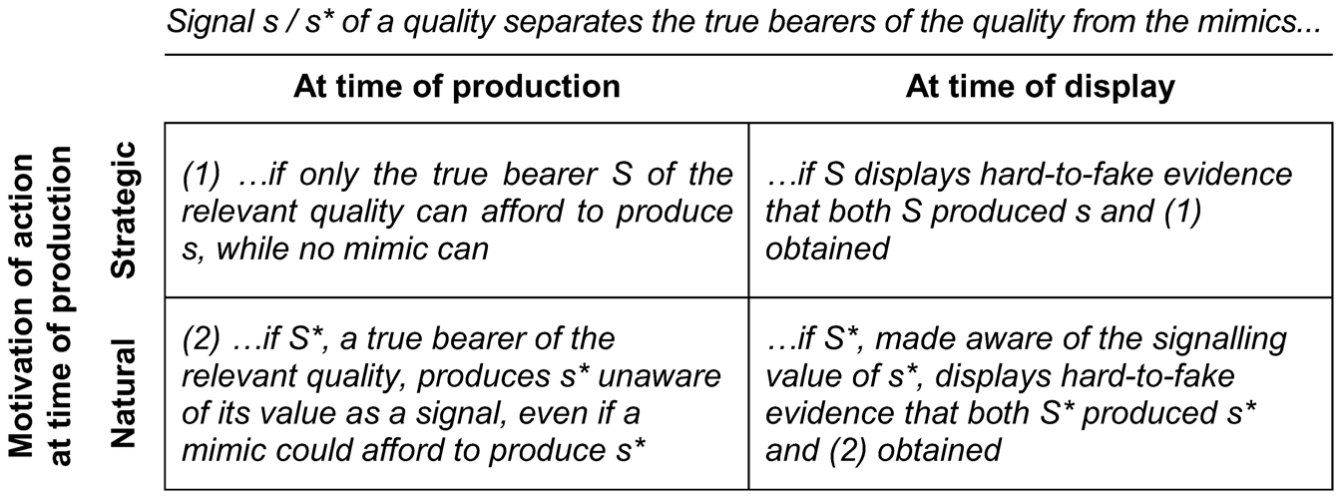
Conceptual diagram. Some actions are strategic, chosen for the purpose of sending information about our qualities to others, to persuade them to act in a way that benefits us; these strategic signals are persuasive if their production is unaffordable by mimics (1). Other actions, chosen for other purposes, can still send information as a by-product; these “natural” signals, or signs, can be persuasive even if their production is cheaper, and could be afforded by mimics (2). Once produced, signals may also be later re-displayed with efficiency gains. This requires hard-to-fake evidence (itself another signal) to prove their production occurred and the “separating” conditions under which it occurred.

Our notion of naturally produced signals is related to Spence's notion of indices ([1]: 9–11 and Ch. 4) and Frank's notion of passive signals ([3]: Ch. 5) in that once an action has been done it cannot be undone. However, by introducing the distinction between signal production and display, indices or passive signals too can be subject to manipulation at the level of display – they can be concealed and denied, but also advertised. Figure 1 summarizes our theoretical argument in a conceptual diagram.

**Figure 2:**
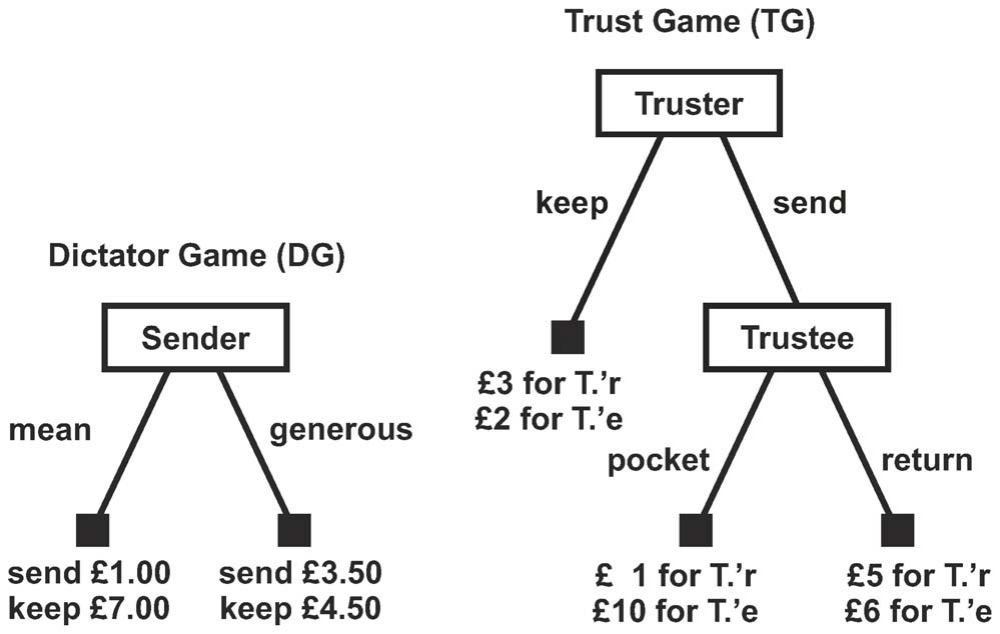
Experimental games. The DG measures subjects' generosity and the TG measures subjects' trust and trustworthiness. In the DG, a sender divides £8.00 between himself and a recipient by choosing “mean” or “generous”. If the sender chooses mean, the recipient receives £1.00 and the sender keeps £7.00. If the sender chooses generous, the recipient receives £3.50 and the sender keeps £4.50. In the TG, the truster is endowed with £5 and decides first between “keep” and “send”. If the truster keeps, the game ends and the truster receives £3 and the trustee £2. If the truster sends, £6 are added to the £5 and the trustee gets to decide between “pocket” and “return”. If the trustee pockets the truster receives £1 and the trustee £10. If the trustee returns, the truster receives £5 and the trustee £6.

In order to investigate the effect of information generated by previous actions on future interactions we use a trust game [4], [5] (TG, Figure 2). In the TG, the primary dilemma for a truster is to decide whether the trustee will resist the temptation to act on his “raw” payoffs and, all things considered, behave trustworthily. If the truster solves that dilemma, then the choice of whether or not to trust follows trivially. But how does he solve it? A common way in which a truster can decide whether a trustee is trustworthy, occurs every time the trustee can choose (or be asked) to emit a signal of his trustworthy-making qualities and the truster can observe it. The truster is reassured if the signal is type-separating. That is, no one without those qualities could afford to emit it [1], [6], [7], [8].

In our computerised laboratory experiment, we give trustees the opportunity to produce such a signal of trustworthiness. The signal is produced by choosing a “generous” over a “mean” split in a dictator game (DG, Figure 2). We assume that a preference for a more equal distribution is linked to the dispositions that incline one to be trustworthy and thus expect generosity to be correlated with trustworthiness. We call the more equal split in the DG “generous”, although it need not be motivated by generosity or altruism, but could be motivated also by inequality aversion or respect of a social norm for sharing—what matters is that all these motives too can sustain trustworthiness. We let subjects decide naturally whether or not to be generous in the DG, by not letting them know that a TG will follow, and compare it with the situation in which they know everything from the start and can choose to be generous strategically. Moreover, before trusters decide in the TG, we let trustees either truthfully reveal (or conceal) or cheap talk (and possibly lie) about how they decided in the DG, and compare it with the situation in which trustees have no possibility to communicate.

Our experiment speaks to a broad range of behavioural topics such as preferences for equity and for truth-telling, and the role of others' intentions in decision making. It is a first attempt to capture a “game” we play in everyday interactions in which we display marks that we have (or pretend to have) naturally acquired simply going about our lives, to honestly (or dishonestly) convince our counterpart of our trustworthiness.

### Previous research

There is good evidence that generosity (including charitable giving) and trustworthiness are correlated [9], [10], [11], [12], [13], that generous individuals are trusted more [11], [13], [14], [15], and that acts of generosity are being used as signals of trustworthiness in social exchange when information about a trustees' reputation is unavailable [13], [15].

Acting trustworthily can be rational in so far as being trusted in the future may pay off more than abusing someone's trust and being distrusted henceforth (e.g. [4], [16], [17], [18], [19] and [20]: 574). Thus, actors can build a reputation for being trustworthy out of self-interest, but a good repute can also emerge as a by-product of their behaving trustworthily out of genuine other-regarding motives. In our experiment, we exclude the possibility of strategic reputation-building by implementing a one-shot game design. In a one-shot TG, self-regarding trustees have no incentive to be trustworthy and therefore, a generous split in the DG should be a signal of trustworthiness due to other-regarding preferences only [13].

Depending on payoffs, a generous split in the DG can still be strategic though. Self-regarding trustees could feign trustworthiness by acting generously with a view to lure trusters to trust them and exploit them to greater gain later on. Empirical evidence indicates that subjects are more cooperative [21] and more generous [22] if they know that their acts will be communicated to a new interaction partner in a subsequent game as compared to a situation in which information about their behaviour is not transmitted. Even mere cues of being watched have been shown to induce more generosity and cooperative behaviour both in laboratory and field experiments [23], [24], [25] (although see [26]). In our experiment, we compare behaviour in a situation allowing for such strategic considerations with a situation in which subjects are not informed that how they decide in the DG could matter later.

Moreover, there is also evidence that verbal non-binding commitments (i.e. “cheap talk”) can be indicative of a trustee's inclination to be trustworthy in a TG [27], [28]. If trustees feel guilty after abusing trust and even more if they have promised otherwise, a promise not to let someone down can be conceived as a “hostage” entailing emotional costs in case trust is abused (although see [29]). However, if the possibility to make such commitments is restricted to the texting of one of the possible actions of the trustee (e.g. “keep”, “split” or blank), it does not convince trusters to trust more than in the control [19]. Rather than letting trustees cheap talk about what they will do in the TG, in one of our experimental conditions we let trustees cheap talk about what they naturally did in the DG (see also [30]).

Finally, there is evidence showing that natural generosity by senders who do not know that their generous act could be reciprocated by the recipient (and the recipient knows that the senders do not know), induces higher reciprocity than generosity that could be strategically motivated because senders know that the recipient can reciprocate [31]. In our experiment, we investigate whether a sender's natural generosity towards a recipient (as opposed to potentially strategic generosity) induces a third party to trust senders more in a trust game.

The next section describes our experimental design and procedure in detail and states our hypotheses. The results section presents our results and the discussion section discusses and concludes. The online Supporting Information (SI) File S1 contains a formal derivation of our hypotheses based on the other-regarding preferences model by Fehr and Schmidt [32]. The SI File S1 also contains the experimental instructions for one of the treatments, shots of the main decision screens, frequency tables referred to throughout the results section, and the regression model estimations on which reported statistics and figures are based.

## Materials and Methods

### Ethics statement

This research was reviewed and approved by the Nuffield Centre for Experimental Social Sciences (CESS) Ethics Review Committee. The experiment was conducted in accordance with CESS Ethical Guidelines. All subjects had given written informed consent before participating in our experiment (see http://cess-web.nuff.ox.ac.uk/experiments/for-researchers/). The anonymised data from this study is available from the authors on request.

### Design

We employ four conditions to capture the essence of what we have described. In each condition we first let subjects decide the split they prefer in the DG and thereafter let them decide in the TG, once in the role of trusters and once in the role of trustees (see Table 1). In the *control* condition and in two other conditions, subjects make their DG choice unaware of what will follow—we call these conditions “veiled”. In the control condition subjects cannot communicate after the DG, but in the other two veiled conditions trustees can inform trusters of their DG choice before trusters decide whether to send them money or not. They can do so in two ways: in one condition (*disclose veiled*), trustees can either conceal or reveal what they truly did in the DG; in the other condition (*declare veiled*), they can simply say what they did in the DG. With these two conditions we reproduce a “natural” situation in which subjects ignore that their DG choice can later acquire an instrumental signalling value in the TG. While in the declare veiled condition they can choose to lie and not only to be silent (as people can do in their CVs for instance), in the disclose veiled condition they implicitly rely on the experimenter to certify that, if they choose to reveal anything, they reveal the truth (this condition approximates the situation in which people rely on a certifying authority). Finally, in the fourth experimental condition (*disclose unveiled*), trustees have the option either to conceal or reveal what they truly did in the DG. The difference to the disclose veiled condition is that we tell subjects from the beginning that a TG with prior communication follows after the DG; they can thus become aware that they can use their choice in the DG as a sign of how generous they are. In this manner we reproduce a case in which DG choices can be made for strategic reasons rather than just because one is more or less generous.

**Table 1:** Experimental design.

	Stage 1	Stage 2	Stage 3
Experimental conditions	Dictator game (DG)	Communication	Trust game (TG)
control (veiled)		Trustees cannot communicate their DG choices to trusters before the TG	Trusters make their TG choices not knowing what trustees' DG choices were
disclose veiled	Senders make their DG choices naturally, ignorant of what stages 2 and 3 comprise	Trustees can truthfully reveal or conceal their DG choices before the TG	
declare veiled		Trustees can say (and possibly lie) or be silent about their DG choices before the TG	Trusters make their TG choices conditional on what trustees communicate their DG choices were
disclose unveiled	Senders can make their DG choices strategically, because they know what stages 2 and 3 comprise	Trustees can truthfully reveal or conceal their DG choices before the TG	

*Notes*: The experiment comprised ten sessions, in each of which up to 30 subjects participated (265 in total). Each session comprised three stages: a DG, a communication stage and a TG. In each session, five subjects were the recipients in the DG (50 in total) and did not participate in stages two and three. The other subjects (215 in total) were assigned to one of four experimental condition: control (veiled), disclose veiled, declare veiled and disclose unveiled. In the three veiled conditions, subjects were informed only after the DG what parts two and three of the experiment comprised. In the unveiled condition it was known to subjects from the beginning what parts two and three would comprise (also see the SI File S1).

### Procedure

We conducted ten experimental sessions. At the beginning of each session, five subjects (50 in total) were randomly selected to be recipients and the other subjects (215 in total) were the senders in the DG (we discuss the implications of this design choice at the end of Section S1 in the SI File S1). The recipients were asked to wait in a different room while senders were randomly assigned to a computer in the laboratory. The instructions given to senders in one of the experimental conditions are reproduced in figures S1 through S4 in File S1. Subjects also had to answer a quiz about the instructions. After answering the quiz, all correct answers were explained to all subjects at the same time by the experimenter. We used the quiz to make sure that subjects understand the experimental set-up and know that every other subject also understands it.

In the first stage of the experiment (see Table 1), the senders in the DG could choose between a mean and a generous allocation of a given amount of money between themselves and a recipient (all decision options were labelled neutrally; see Figure S5 in File S1). As soon as all senders had made their decisions, as many “mean” and “generous cards” as there were mean and generous choices made by the senders were put in an opaque box. Recipients determined their payoffs by privately drawing one card from the box, were paid accordingly and left. The senders remained in the laboratory and were randomly assigned to one of the three veiled conditions. The disclose unveiled condition was tested separately as it was not possible to segregate subjects in such a way as to conduct the veiled and unveiled conditions in the same session.

In the third stage, each subject played the TG twice, once as a truster and once as a trustee. In each session, all subjects but one were randomly assigned to play the TG as a trustee first. We wanted subjects in the TG to take the trustee decision before the truster decision because we wanted to make it as easy as possible for subjects to see the TG from a trustee's perspective when deciding as a truster. Subjects knew that they would be paired with two different subjects from the same session in order to determine the outcomes of both trust games and that they would receive feedback about these outcomes only at the end of the experiment.

We used the strategy method to elicit subjects' decisions in the TG. That is, trusters had to decide between keeping and sending conditional on what trustees had revealed or stated in the second stage about their decision in the DG in the first stage (see figures S6 and S7 in File S1). Accordingly, trustees had to decide between pocketing and returning for the case in which a truster chose to send (see Figure S8 in File S1). Although using the strategy method could lead to underestimate trustworthiness [33], this should not weaken the ability of our findings to establish whether our theoretical considerations are supported or not (see [34]).

Subjects were Oxford University students, 46% were female and they were 23.4 (sd = 4.91) years old on average. A subject's total earning was the sum of what they earned in the DG as a sender, in the TG as a truster and as a trustee, and their show-up fee of £4. After the experiment, subjects filled in a questionnaire, were paid and left the lab. An experimental session lasted about 75 minutes and subjects who participated in the entire session earned £17.91 on average. The experiment was programmed and conducted with the software z-Tree [35].

### Hypotheses

Based on our theoretical considerations in the SI and the empirical evidence we referred to in Section 1.1, we expect generosity in the DG to be correlated with trustworthiness in the TG (Hypothesis H1). Moreover, we expect that generous trustees will choose to inform trusters truthfully, while mean trustees will be silent or, when given the opportunity in the declare condition, lie and state that they were generous when they were not (Hypotheses H2a, H2b and H2c). That is to say, we expect subjects to be able to see the strategic value of their DG choice irrespective of whether they made it when they were aware or not aware of what was to follow, and act accordingly. Also regardless of treatment, trusters should “send” more often to trustees who disclose or declare that they were generous than to trustees who remain silent or disclose or declare that they were mean (Hypotheses H3a, H3b and H3c). In the disclose condition, but not necessarily in the two other treatment conditions (see below), trusters should also send more often to trustees who disclose that they were generous than to trustees about whom they have no information as in the control condition (Hypothesis H4a).

The unveiled treatment elicits deeper strategic behaviour. It encourages subjects, who would be naturally mean, to pay a signalling cost and be generous in the DG, to then be able to disclose it to trusters and be more likely to obtain their trust. The rewards of switching from mean to generous in the DG depend on whether the trustee plans to be trustworthy or untrustworthy in the TG. If the trustee plans to be untrustworthy, there is a net gain from switching if, after being informed that the trustee was generous, a truster would trust him with more than 31% probability; for a trustee who plans to be trustworthy a net gain accrues only if this probability is above 63% (this holds for our payoffs of course, and assuming that no truster would trust a trustee who was mean and disclosed it or remained silent). Those who switch strategically are then likely to be mainly the untrustworthy subjects.

This implies that the proportion of generous subjects should be higher than in the veiled conditions (Hypothesis H0c). As a result, the correlation with trustworthiness should be lower in the unveiled condition—generosity, in other words, should become a less reliable signal of trustworthiness (Hypothesis H1c).

Correspondingly, those trusters with their strategic heads firmly in place should discount information coming from trustees in the treatments that are potentially mimic-beset. That is, compared to the control condition, they should be no more likely to trust trustees who can merely say that they were generous and where this could be a lie (Hypothesis H4b), and trustees in the unveiled case, in which information is true, but could be designed as a bait to induce trust and abuse it (Hypothesis H4c). Table 2 lists all our hypotheses. How these hypotheses are formally derived can be seen in the SI File S1.

**Table 2:** Hypotheses.

**All conditions**	
**H1:**	An actor who chose generous in the DG, when playing the TG as a trustee, will be more likely to choose return than an actor who chose mean in the DG.
**Disclose veiled condition**	
**H2a:**	An actor who chose generous in the DG, when playing the TG as a trustee, will be more likely to reveal his or her choice to the truster than an actor who chose mean in the DG.
**H3a:**	A truster in the TG will be more likely to choose send if the trustee truthfully revealed that in the DG his or her choice was generous than had the trustee revealed that his or her choice was mean or had he or she concealed his or her choice.
**H4a:**	A truster in the TG will be more likely to choose send if the trustee truthfully revealed that in the DG his or her choice was generous than when he or she has no information about the trustee as in the control condition.
**Declare veiled condition**	
**H2b:**	An actor who chose generous in the DG, when playing the TG as a trustee, will be more likely to reveal his or her true choice than an actor who chose mean in the DG.
**H3b:**	A truster in the TG will be more likely to choose send if the trustee said that in the DG his or her choice was generous than had the trustee said that his or her choice was mean or had he or she remained silent.^a^
**H4b:**	A truster in the TG will discount any information about what the trustee said that his or her choice was in the DG and will be equally likely to choose send as in the control condition, in which he or she has no information about the trustee.
**Disclose unveiled condition**	
**H1c:**	The correlation between generous choices in the DG and return choices in the TG will be smaller than predicted under H1.^b^
**H2c:**	An actor who chose generous in the DG, when playing the TG as a trustee, will be more likely to reveal his or her choice than an actor who chose mean in the DG.
**H3c:**	A truster in the TG will be more likely to choose send if the trustee truthfully revealed that in the DG his or her choice was generous than had the trustee revealed that his or her choice was mean or had he or she concealed his or her choice.
**H4c:**	A truster in the TG will discount the information about the second mover truthfully revealing that in the DG his or her choice was generous and will be less likely to choose send than predicted under H4a.

a Hypothesis H3b does not follow from our theoretical argument according to which all trustees will say that they chose generous in the DG. However, should trustees who chose mean in the DG tell the truth, we expect trusters to trust them less than trustees who say that they chose generous.

b Hypothesis H1c presupposes that the proportion of generous subjects should be higher than in the veiled conditions (Hypothesis H0c).

## Results

Overall, out of our 215 subjects, 23% chose generous in the DG. This is comparable to DG choices student subjects made in an experiment that was ran by different researchers in the same lab two years before ours [36]. In line with our assumption, a generous choice proves to be an accurate sign of trustworthiness in the TG. Eighty per cent of the generous subjects chose return in the TG, while only 28% of the mean subjects did (H1: χ^2^_(1)_ = 42.27, *p*<0.001). The 20% who chose generous in the DG and then chose pocket in the TG we expect to be the strategic trustees. The subjects who were mean in the DG but then chose return in the TG are interesting because they suggest that generosity is not the only motive for trustworthiness. One can be ungenerous in distributing sums of money upon which one has control but still trustworthy either because of reciprocity [37] or trust-responsiveness, that is, an obligation to live up to the trust which is put into one [38]. Still, we cannot be sure for their return choice could be somehow meant to make up for their first one.

### Trustees' communication strategies

**Figure 3:**
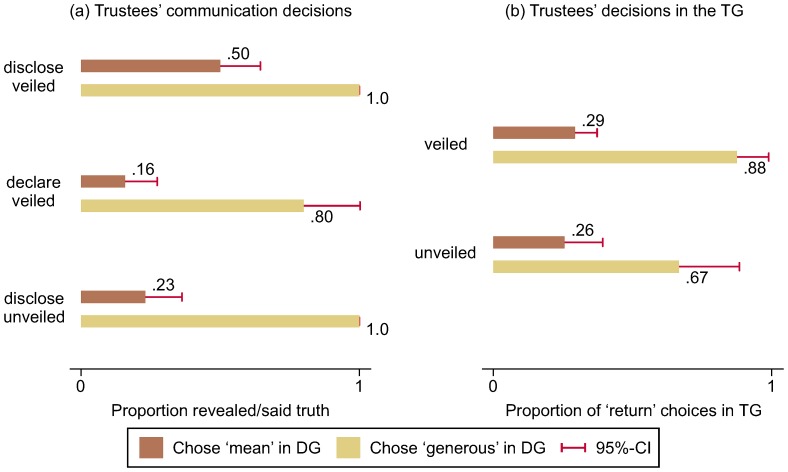
Trustees' behaviour in the communication stage (a) and in the TG (b) conditional on their DG choices. The overall rate of trustees who chose return in the TG is 40% (and 40% in the control condition only). This is in line with what Snijders and Keren [39] find with Dutch students (44%) and what Bolton and colleagues [40] find with German students (37%), but lower than what McCabe and colleagues [37] find with US students (65%). However, McCabe and colleagues [37] give trustees a comparatively high monetary incentive to choose return and their rate is similar to what Snijders and Keren [39] find with a comparable payoff structure (63–75%). In our study, moreover, mean trustees are less likely to truthfully reveal or say before the TG what they did in the DG (a) and are also less trustworthy than generous trustees (b) (also see Table S1 in File S1).

Trustees' decisions in the DG have a powerful effect in determining their choice of whether to inform trusters. Across all experimental conditions, virtually *all* generous trustees chose to inform trusters truthfully (93%), whereas only 31% of mean trustees did (H2: χ^2^_(1)_ = 46.12, *p*<0.001; see Figure 3a for the disaggregated numbers). This suggests that trustees of both types realised the value of their choice in the DG, and acted accordingly.

When we look only at mean trustees and at their choices of whether and how to inform trusters in the different treatments (Figure 3a), some interesting results emerge. Mean trustees are more likely to reveal their choice to trusters in the disclose condition than in either the declare condition (χ^2^_(1)_ = 9.84, *p* = 0.002) or the unveiled condition (χ^2^_(1)_ = 6.26, *p* = 0.012). It seems as if mean trustees feel more at ease confessing their “sins” when they did not know what was to follow, *and* when silence is their sole alternative to telling the truth. This result cannot be explained by a preference for honesty or lying aversion [41] because when lying is an option, there is a massive shift in trustees' communication strategies. Only 16% tell the truth, 24% keep quiet and 60% lie declaring that they were generous when they were not. Finally, consistent with Hurkens and Kartik [42], lying appears neither idle nor emotionally driven. While 83% of the truth-tellers (15 out of 18) in the declare condition, whether mean or generous, went on to be trustworthy (χ^2^_(1)_ = 15.33, *p*<0.001), 78% of the liars (18 out of 23) went on to be untrustworthy. The expected payoffs of those who were mean in the DG, lied about it and were untrustworthy turn out to be the highest (see Table S1 in File S1). The remaining 12 trustees in the declare condition were silent. Nine of them had been mean and three had been generous. While all three of those who had been generous and silent were trustworthy, only three of the mean ones (33%) were also trustworthy. This difference is statistically significant (χ^2^_(1)_ = 4.00, *p* = 0.046).

### Trusters' responses to trustees' signals

Across treatments between 47% and 55% of trusters are unresponsive, and either trust everyone or no one regardless of the information they receive. A sizeable share of trusters however perceive generosity as a signal of trustworthiness and meanness as one of untrustworthiness, and respond by trusting only those who inform them that they were generous in the DG. Nineteen per cent of trusters respond in this way in the declare condition in which subjects can lie, and 34% and 40% respectively in the disclose veiled and unveiled conditions in which subjects cannot lie. The significantly lower proportion of conditional trusters in the declare condition is a first indication that trusters are less inclined to trust in response to a declaration of generosity which could be a lie (χ^2^_(2)_ = 6.16, *p* = 0.046). This suspicion seems justified as conditionally trusting is the strategy with the highest expected payoffs in the disclose and the unveiled conditions, while in the declare condition it yields the lowest expected payoff (see Table S2 in File S1).

Trusters' response to information has two effects on the aggregate level. First, the overall level of trust is substantially higher when trusters receive positive rather than negative information. If a trustee revealed or said that they had been generous, 65% of the trusters chose send, whereas if a trustee revealed or said that they had been mean or remained silent about their decision in the DG, only 29% of the trusters chose send (H3: χ^2^_(1)_ = 73.99, *p*<0.001). Second, positive information significantly increases the overall level of trust relative to the control condition, in which 46% of the trusters chose send without knowing anything about the trustees (χ^2^_(1)_ = 5.72, *p* = 0.017). Correspondingly, negative information reduces trust significantly as compared with the control condition (χ^2^_(1)_ = 5.11, *p* = 0.024).

### Trustees' mimicking trustworthiness

We hypothesise that in the unveiled condition more subjects would be generous strategically, with a view to lure trusters to trust them in the TG and maybe to exploit them (recall that in the unveiled condition subjects cannot lie to trusters about their DG choice, but only reveal it or be silent). In fact, and in line with previous findings [22], the proportion of senders who chose generous in the DG is higher in the unveiled condition (32%) than in the veiled conditions (20%) though the difference is significant only at the 10% level (H0c: χ^2^_(1)_ = 3.01, *p* = 0.083). The limited size of the difference can result from a second order reasoning. Trustees may have thought that since trusters know that the signal is mimic-beset, trusters will be likely to disregard it and less likely to trust even a generous trustee. Consequently, some trustees may have concluded that it does not pay off to pretend to be generous in the DG.

But is generosity a murkier signal of trustworthiness in the unveiled than in the veiled condition? While the correlation between subjects' decisions in the DG and the TG is purer in the veiled conditions than in the unveiled condition (Figure 3b), the difference in differences is not significant (H1c: χ^2^_(1)_ = 1.60, p = 0.205). Thus, we fail to find evidence in favour of Hypothesis H1c.

The aggregate data in Figure 3b, however, do not reveal to what extent unveiling the second part triggers strategic generosity in the first part. To find out we need to consider the behaviour of individual trustees across their DG and TG choices (see Table S1 in File S1). This analysis reveals that there are only 10 trustees (5%) whom we could call “strategic” to describe the fact that they were generous in the DG and went on to pocket in the TG, but, in line with our expectation, we find a significantly higher proportion of them in the unveiled condition (11%) than in the veiled conditions (3%) (χ^2^_(1)_ = 6.04, p = 0.014). In other words, informing subjects from the start that a TG will follow the DG triggers strategic generosity, but only in a small proportion of subjects.

### Trusters' response to mimic-beset signals

Our conjecture is that clever trusters would suspect that untrustworthy trustees might try to mimic trustworthy ones either at the level of the signal display (by lying at the communication stage) or at that of the signal production (by pretending generosity in the DG). To find out we need to look at trust levels separately for the three treatments.

**Figure 4:**
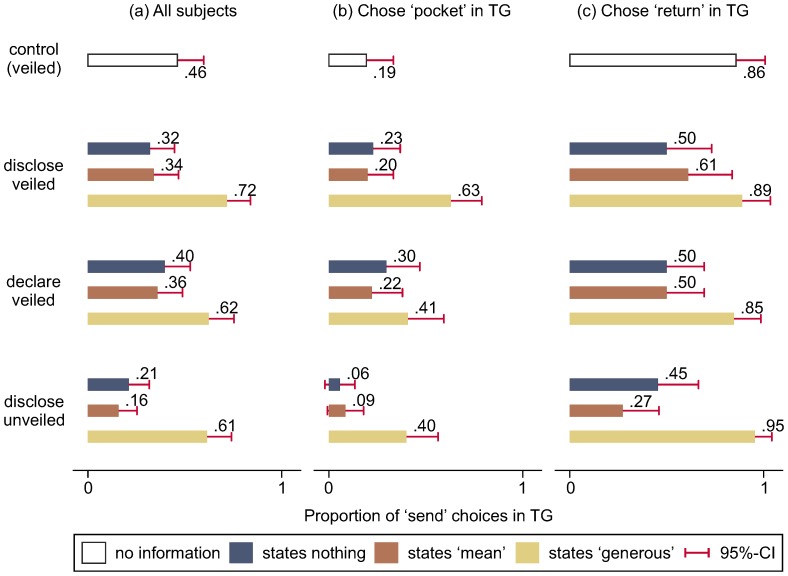
Trusters' behaviour in the TG conditional on trustees' communication decisions. Trusters are more likely to trust trustees who state that they were generous (65%) than they are likely to trust trustees about whom they have no information (46%) or who remain silent or state that they were mean in the DG (29%) (a). Trusters who themselves were untrustworthy are more likely to discount mimic-beset positive information (b) than trusters who were trustworthy as trustees in the TG (c) (also see Table S2 in File S1).

Figure 4a shows the proportion of trusters who chose send in the TG across experimental conditions and contingent on the information they received from the trustees. It is apparent, as we pointed out already, that in all treatment conditions trusters trust substantially more in response to positive information than negative information—this they do *irrespective* of the type of negative information. Whether a trustee revealed or just said to have chosen mean in the DG or did not reveal or say anything, does not make a difference (χ^2^_(3)_ = 2.23, *p* = 0.527). For trusters, confession does not seem to signal remorse, nor is it perceived as better than silence and thus cannot be interpreted as a tacit apology [30], [43]. Trustees who disclose that they were mean when they knew from the start that a TG was going to follow (unveiled condition) are the least trusted of all; they are trusted significantly less than trustees who revealed that they had been mean in the disclose and declare conditions (χ^2^_(1)_ = 5.01, *p* = 0.025 and χ^2^_(1)_ = 6.00, *p* = 0.014, respectively). It seems as if to be self-regarding is bad and to shamelessly confess it worse.

More importantly, as we expected, the proportion of sending choices given positive information is significantly higher in the disclose condition than in the control condition (H4a: χ^2^_(1)_ = 6.86, *p* = 0.009). But positive information does not have the same significant effect in the declare (H4b: χ^2^_(1)_ = 2.71, *p* = 0.100) and in the unveiled condition (H4c: χ^2^_(1)_ = 2.51, *p* = 0.113). Trusters, in other words, are not significantly more inclined to trust when they know that the generous choice is just stated or that it could have been made strategically than when they know nothing of the trustee as in the control condition. This implies that information which could be mimic-beset is not significantly better than no information at all. However, the differences in differences are statistically insignificant (χ^2^_(1)_ = 1.06, *p* = 0.304 and χ^2^_(1)_ = 1.29, *p* = 0.256, respectively).

The evidence that subjects discount positive information in the declare and the unveiled condition becomes clearer and stronger once we isolate trusters who are more likely to be suspicious of others from trusters who are more likely to be laid back and not to think that others are scheming at their expense. To do so, we analyse separately the responses of trusters who *as trustees* chose pocket and those who chose return in the TG. We assume that those who themselves are untrustworthy will be alert to the possibility of others either lying in the declare condition or just pretending to be generous in the DG in anticipation of the TG.

Figures 4b and 4c show the separate results for “wary” and “relaxed” subjects, respectively. First, confirming previous evidence on this link (e.g. [44], [45]), these figures reveal a strong positive correlation between trustworthiness and trust: the difference in trust levels in the control condition amounts to a staggering 67 percentage points (0.19 vs. 0.86). Most importantly, in the light of positive information, relaxed subjects' propensity to trust does not differ across treatments whereas wary subjects (who chose pocket as trustees in the TG) trust most in the disclose condition (63%). They trust less if what the trustee states could be a lie (41%) (χ^2^_(1)_ = 2.92, *p* = 0.088) or, although truthfully stated, could have been produced in bad faith (40%) (χ^2^_(1)_ = 3.57, *p* = 0.059).

## Discussion

The subtlety and variety of our communications, in which we try, and at times fail to control the image we project or to uncover the true nature of others lying beneath that image—a terrain so richly described by Erving Goffman [46],[47]—is at present largely beyond the reach of formal theorizing. Here we try to extend the reach of signalling theory, which, despite the appeal of its rigorous and elegant simplicity, has remained a rather abstract and blunt instrument. In our effort, we have explored the case in which we employ acts we produced for some other purpose independent of signalling, to signal something about us. When we become aware of the information value of our past actions, we display (or conceal) evidence of what we did. What was a dormant sign, becomes, by being displayed, an intentionally emitted signal.

Signal display can exploit sign production efficiently, but the reliability of the signal display is not coextensive with that of its production. Observing production allows us to infer something about the unobservable properties of the signaller—there is a causal connection between the signal and the property—but this is not so in the case of the display. Still, the theory's principles can be applied not only to what we do, but to the evidence that we did it.

We set up a laboratory experiment involving a dictator game and a trust game to test the empirical implications of the distinction between signal production and display. We exploit the fact that choosing a generous split unconditionally is closely associated with being trustworthy, a link so strong as to suggest that both behaviours are driven by overlapping dispositions.

Subjects understand this link and use it appropriately in their communications. In the TG, trusters adjust their trusting decision to the information they receive on what trustees' distributive choices in the DG were; and trustees try to persuade trusters to trust them by revealing (or concealing) their distributive choices—to act in this way, trustees must understand that trusters too understand.

Subjects understand the link between generosity and trustworthiness so well in fact that when they are not generous, they mostly lie about it. Moreover, while a great number of those who tell the truth, even if they chose mean in the DG, go on to be trustworthy—we found great consistency in “badness”: trustees who are mean are much more likely not to tell the truth to trusters, and trustees who do not tell the truth are much more likely to be untrustworthy.

Lying apart, there are other indications that subjects can be strategic in how they handle information whether emitted or received. Although few in number, we find significantly more subjects choosing generous but then pocketing the sums that trusters pass on in the unveiled condition, in which they know that their choice in the DG could become valuable information in the TG, than in the veiled conditions. In other words, removing the “veil of ignorance”—when instrumental considerations can inspire the choice and hence mar its information value—tends to cloud the link between generosity and trustworthiness.

But trusters are not all gullible. They rely significantly more on the positive information they receive from a source that trustees cannot manipulate, while they are more careful when the trustees could either lie about or just pretend being generous. This is especially true for those trusters who are themselves untrustworthy when they decide as trustees.

It is striking to uncover such indications of strategic thinking even in an artificial setting with low stakes. It thus seems natural to surmise how much more intense the strategizing around these decisions and their presentation must be in domains more extreme than our lab, in which stakes are high and mistakes paid dearly.

But why is natural generosity not crowded out by strategizing? When stakes are small at least, it seems as if even mimics “relax”—their bounded cognition prevents them from being strategic all the time [48]. This allows natural generosity to remain distinguishable from instrumental generosity, with the efficiency advantage of producing credible information about our other-regarding preferences at low cost. Such information, in turn, reduces uncertainty in social interactions and establishes favourable conditions for gains to be made from mutual cooperation [13].

Everyday interactions in most human groups offer opportunities to share resources with others, and simple, easily observable acts of sharing (or not sharing), give an indication of actors' unobservable but relevant traits [49], [50] such as other-regarding preferences. Thus, acts of generosity may have arguably been the first signs of reliability in cooperation, from which actors were able to infer others' trustworthiness [13], [51], [52]. In mutually beneficial interactions trustworthy actors are more attractive partners than opportunists are, thus generosity as a signal of trustworthiness should spread through positive assortment [53] and partner choice [54], all the more as generous acts are signals that provide benefits to others [55], [56].

## Supporting Information

File S1 **Supporting file that contains all supporting information that is referred to throughout the article.**
(PDF)
