# Supplementary material for: Natural and Strategic Generosity as Signals of Trustworthiness
Source: PLoS One. 2014 May 15;9(5):e97533. doi: 10.1371/journal.pone.0097533 (PMC4022519; doi:10.1371/journal.pone.0097533)
Supplement: File S1 — Supporting file that contains all supporting information that is referred to throughout the article. (PDF) [file pone.0097533.s001.pdf]

Supporting Information (SI)

Natural and strategic generosity as signals of trustworthiness

Diego Gambetta[ab] and Wojtek Przepiorka[bc†]

[a] European University Institute, Department of Political and Social Sciences, San Domenico di Fiesole (FI), Italy

[b] Nuffield College, Oxford, United Kingdom

[c] University of Oxford, Department of Sociology, Oxford, United Kingdom

[†] Author for correspondence: wojtek.przepiorka@sociology.ox.ac.uk

S1. Hypotheses

We use the Fehr and Schmidt model [S1] (henceforth F&S model) to derive our hypotheses. The F&S model is very tractable in this setup and allows us to give clear intuitions to readers. It is not our aim to test the point predictions of the F&S model; based on plausible assumptions, we use it to derive behavioural hypotheses that can be confronted with the results of our experiment, as well as with common sense and our intuitions. The F&S model has been contested in its empirical [S2] and methodological validity [S3,S4,S5], and there are other models of other-regarding preferences which could be used instead (e.g. [S6,S7,S8]). These however would, in all likelihood, yield qualitatively similar results.

In the F&S model, the utility of agent i at a terminal node of a two-person extensive form game is:

$$u_i = \pi_i - \alpha_i \max(\pi_j - \pi_i; 0) - \beta_i \max(\pi_i - \pi_j; 0) \quad \text{(S1)}$$

Actor i's (monetary) payoff is denoted by π_i, α_i is the so called "envy" parameter, and β_i the so called "guilt" parameter. It is assumed that $\alpha_i \geq 0$, $\alpha_i \geq \beta_i \geq 0$ and $\beta_i < 1$. In other words, no actor i likes having less than actor j, no actor i likes having more than actor j but dislikes having less more than he or she dislikes having more, and actor i's dislike for having more never outweighs his or her utility from having π_i, respectively. Furthermore, it is assumed that agents have private information about their other-regarding preferences α and β, and are ignorant of the probability distribution of α and β in the population.

Taken to our experimental games (see Figure 1), an agent i will choose generous in the DG, if $4.5 - \beta_i(4.5 - 3.5) > 7 - \beta_i(7 - 1)$, thus, if $\beta_i > 0.5$; the same holds for an agent i moving second in the TG. This suggests that generosity in the DG will be highly correlated with trustworthiness in the TG.

H1: An actor who chose generous in the DG will be more likely to choose return as a second mover in the TG than an actor who chose mean in the DG.

Control condition

What will an F&S-rational agent *i* as a first mover in the TG do? Let *p* be the probability with which agent *i* expects to interact with a trustworthy second-mover *j*, i.e. $p = \Pr[\beta_j > 0.5 \mid j]$. Agent *i*'s expected utility from choosing send in the TG is $p[5 - \alpha_i(6 - 5)] + (1 - p)[1 - \alpha_i(10 - 1)]$ and agent *i*'s utility from choosing keep is $3 - \beta_i(3 - 2)$. If the former is larger than the latter, that is, if $p > (2 + 9\alpha_i - \beta_i) / (4 + 8\alpha_i)$, then agent *i* will choose send and will choose keep otherwise. For $\alpha_i = 0.7$ and $\beta_i = 0.5$ – values well within the range estimated by Blanco and colleagues [S2] – agent *i* would have to expect to interact with a trustworthy *j* with a probability higher than 0.812 in order to choose send.

Disclose veiled condition

If agent *j*, before agent *i* decides whether to send or to keep in the TG, truthfully revealed that in the DG his or her choice was generous, agent *i* would know with certainty that agent *j* is trustworthy and choose send (except for relatively high values of α_i or α_i and β_i). Accordingly, if agent *j* truthfully revealed that in the DG his or her choice was mean, agent *i* would know with certainty that agent *j* is not trustworthy and choose keep. Since only an untrustworthy *j* could gain from concealing his or her choice in the DG, and thus by doing so would reveal his or her type, an untrustworthy *j* should be indifferent between truthfully revealing and concealing his or her mean choice in the DG. A trustworthy *j* will always reveal his or her generous choice.

H2a: An actor who chose generous in the DG will be more likely to reveal his or her choice as a second mover in the TG than an actor who chose mean in the DG.

H3a: A first mover in the TG will be more likely to choose send if the second mover truthfully revealed that in the DG his or her choice was generous than had the second mover revealed that his or her choice was mean or had he or she concealed his or her choice.

H4a: A first mover in the TG will be more likely to choose send if the second mover truthfully revealed that in the DG his or her choice was generous than he or she is likely to choose send in the control condition, where he or she has no information about the second mover.

Declare veiled condition

Actors' incentives change if rather than truthfully revealing or concealing their choice in the DG, second movers in the TG could merely say what they chose or remain silent. Assuming rational agents, those who chose mean in the DG will lie and say that they chose generous instead. Consequently, since all actors will say that they chose generous in the DG, such information will be discarded by the first movers in the TG.

H2b: An actor who chose generous in the DG will be more likely to tell his or her true choice as a second mover in the TG than an actor who chose mean in the DG.

H3b: A first mover in the TG will be more likely to choose send if the second mover said that in the DG his or her choice was generous than had the second mover said that his or her choice was mean or had he or she remained silent.[1]

H4b: A first mover in the TG will discard any information about what the second mover said that his or her choice was in the DG and will be equally likely to choose send as in the control condition, where he or she has no information about the second mover.

Disclose unveiled condition

Finally, if all actors know from the beginning that a TG will follow the DG and that second movers will have the opportunity to truthfully reveal or conceal their choices in the DG before first-movers decisions in the TG, strategic considerations will change actors' incentives.

Now, actor *i*'s decision in the DG can be conceived as a signal and the whole game can be analysed as one of signalling with agents' types being their private information *a priori*. In this game, agent *i* might prefer to forgo part of their payoff in the DG to pass for an agent with $\beta_i > 0.5$. Note that only agents with $\beta_i < 0.5$ would reason strategically for only they will benefit from being mistaken for trustworthy. Agents with $\beta_i > 0.5$ have negative signalling costs and will therefore always prefer the generous over the mean choice in the DG. For agents with $\beta_i < 0.5$, the signalling cost c is the utility difference between the mean and the generous choice in the DG, i.e. $7 - \beta_i(7 - 1) - [4.5 - \beta_i(4.5 - 3.5)] = 2.5 - 5\beta_i$. Thus, for an agent *i* with $\beta_i = 0$, the signalling costs are maximal and go towards zero with β_i approaching

[1] Hypothesis H3b does not follow from our theoretical argument according to which all trustees will say that they chose generous in the DG. However, should trustees who chose mean in the DG tell the truth, we expect trusters to trust them less than trustees who say that they chose generous.

0.5. Clearly, if agent *i* chooses mean in the DG, agent *j* will choose keep in the TG. It is not clear, however, what agent *j* will do if agent *i* chooses generous in the DG. Let *q* be the probability with which agent *j* chooses send in the TG conditional on agent *i* having chosen generous in the DG, i.e. $q = \Pr[a_{j,TG} = \text{“send”} \mid a_{i,DG} = \text{“generous”}]$. Agent *i*’s expected utility from choosing generous in the DG is $q[10 - \beta_i(10 - 1) - c] + (1 - q)[2 - \alpha_i(3 - 2) - c]$ and agent *i*’s utility from choosing mean is $2 - \alpha_i(3 - 2)$. Thus, agent *i* will choose generous in the DG if the former is larger than the latter, that is, if $q > c / (8 - 9\beta_i + \alpha_i)$. Recall that we are only considering agents with $0.5 > \beta_i \geq 0$. For this range of agents *i*, the maximum they have to expect *q* to be for them to mimic a generous type in the DG is 0.313 (if $\beta_i = 0$). It is therefore likely that a significant proportion of agents i with $\beta_i < 0.5$ will choose generous in the DG. However, if agents *j* anticipate such mimicry, they will discount the information of the generous choice and be less likely to send in the TG. Thus, the proportion of generous choices in the DG and send choices in the TG will ultimately depend on agents *i*’s and *j*’s higher order beliefs. Based on what we know about different degrees of strategic sophistication (e.g. [S9]), we may expect the following:

H1c: The correlation between generous choices in the DG and return choices in the TG will be smaller than predicted under H1.[2]

H2c: An actor who chose generous in the DG will be more likely to reveal his or her choice as a second mover in the TG than an actor who chose mean in the DG.

H3c: A first mover in the TG will be more likely to choose send if the second mover truthfully revealed that in the DG his or her choice was generous than had the second mover revealed that his or her choice was mean or had he or she concealed his or her choice.

H4c: A first mover in the TG will discount the information about the second mover truthfully revealing that in the DG his or her choice was generous and will be less likely to choose send than predicted under H4a.

One could object that the theoretical argument outlined above does not exactly match with our experimental set-up. In each session of our experiment, there were between 23 and 30

[2] Hypothesis H1c presupposes that the proportion of generous subjects should be higher than in the veiled conditions (Hypothesis H0c).

participants only five of which were randomly assigned to be the recipients in the DG while the remainder were the senders. Given the experimental procedure (see the instructions in Figure S2 below), a sender's generosity would affect the outcome of a recipient only with a certain probability (and not with certainty as our theoretical argument thus far suggests). In what follows, we extend our theoretical argument to accommodate the case in which the interaction takes place between one sender and five recipients, and the sender's generosity can affect each of the five recipients' outcomes with equal probability. In this scenario, the cost from being generous is the same as before but the expected benefits for the recipient(s) will be lower. Therefore, we can expect that many senders who would be generous in the one-to-one interaction will now refrain from being generous, and only the most other-regarding individuals will still decide to be generous.

In an experimental session with n senders and five recipients, the probability that x out of the five recipients will benefit from one particular sender's generosity is:

$$p(x) = \binom{5}{x}\left(\frac{1}{n}\right)^{x}\left(\frac{n-1}{n}\right)^{(5-x)} \tag{S2}$$

Moreover, in an interaction between one sender i and five recipients j, an other-regarding sender i's utility depends on the outcome of all five recipients.[3] The F&S model accommodates this more general case as follows (see [S1]: 822):

$$u_i = \pi_i - \alpha_i \frac{1}{5}\sum_{j=1}^{5} \max(\pi_j - \pi_i; 0) - \beta_i \frac{1}{5}\sum_{j=1}^{5} \max(\pi_i - \pi_j; 0) \tag{S3}$$

Since in the DG, the sender's payoff is always larger than a recipient's payoff, Equation S3 can be simplified to:

[3] Note that the probability that a sender i's generosity will benefit x out of five recipients (Equation S2) is a function of the number of senders n, while the sender's utility u_i (Equation S3) is not a function of other senders' outcomes. In experiments in which many one-shot interactions take place at the same time (e.g. in one session), the possible outcomes of other groups is typically not considered in the game theoretical analysis.

$$u_i = (1-\beta_i)\pi_i + \frac{\beta_i}{5}\sum_{j=1}^{5}\pi_j \qquad \text{(S4)}$$

Note that π_j is now stochastic. Thus, the sender's utility from choosing mean in the DG is $7(1 - \beta_i) + \beta_i = 7 - 6\beta_i$, and the sender's utility from choosing generous is $4.5(1 - \beta_i) + \beta_i/5\ [5p(0) + 7.5p(1) + 10p(2) + 12.5p(3) + 15p(4) + 17.5p(5)] = 4.5(1 - \beta_i) + 1.114\beta_i = 4.5 - 3.386\beta_i$, where $p(x)$ denotes the probability that x out of the five recipients will benefit from the sender's generosity (see Equation S2) assuming $n = 22$ senders (the session average in our experiment) only one of which (sender i) chooses generous in the DG. In this interaction, the sender chooses generous in the DG if $4.5 - 3.386\beta_i > 7 - 6\beta_i$, that is, if $\beta_i > 0.956$, and chooses mean otherwise.

Although this further analysis shows that our experimental set-up reduces the number of subjects who would be generous in the DG relative the case in which one sender interacts with one recipient only (as it moves the β-threshold for generous behaviour from 0.5 to 0.956), the behavioural hypotheses we can derive based on this theoretical framework remain intact. In the experimental instructions we are clear about the actual procedure with which payoffs are calculated (see Figure S2), but to simplify our prose we describe the interaction as one between one sender and one receiver.

S2. Experimental instructions

CESS Instruction Sheet

Dear participant

Welcome and thank you for participating in this experiment. Please take your time to read through the following instructions thoroughly. You can take notes if you so wish.

Important rules and practical details

- This session will last for about 90 minutes.
- Your participation is voluntary and you are free to leave at any point, if you wish to do so. In that case, we will only pay you the show-up fee of £4.
- We ask you not to communicate verbally or otherwise with the other participants and generally to remain quiet until the end of the session. Those who do not respect the silence requirement may be asked to leave.

What will happen at the end of the session?

Once the session is finished, please remain seated. We will need around 10 minutes to prepare your payment. You will be called up one at a time by the number on your table; you will then receive an envelope with your earnings and you will be asked to sign a receipt.

General information about the experiment

You are participating in an experimental session in which you will earn some money. The money you earn during this session will be added to your show-up fee of £4 and paid in cash at the end of the session. Your earnings depend on both the decisions you will make and the decisions other participants will make. Up to 30 people are participating in this session. All decisions taken in this experiment are anonymous and the data collected in this experiment are for scientific purposes only.

The experiment consists of three parts and the money you earn during this session is the sum of what you earn in each part. We start by giving you instructions on the first part. Please proceed on the screen once you have read the Part 1 instructions. You will then be asked to answer a few control questions about these instructions on the screen. Next, your answers and the correct answers will appear on your screen. The person conducting this session will then read out all control questions and give and explain the correct answers. Finally you will get the opportunity to ask questions. Once all questions are answered, the first part of the experiment will start. The second and third part will proceed in a similar way.

1

Figure S1: Part 1 instructions in the disclose condition (page 1)

CESS Instruction Sheet

Instructions (Part 1)

For the first part of the experiment each participant is randomly assigned to be a "recipient" or a "sender". If you are a recipient, you will not have a possibility to make a choice. If you are a sender, you will be asked to divide £8.00 between yourself and the recipient by choosing between action "h" and action "p" (see Figure 1, the letters "h" and "p" are arbitrary and have no meaning).

- If you choose action "h", the £8.00 will be divided between you and the recipient such that the recipient will receive £1.00 and you get to keep £7.00.
- If you choose action "p", the £8.00 will be divided between you and the recipient such that the recipient will receive £3.50 and you get to keep £4.50.

Figure 1

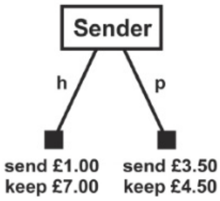

If you are a sender, you will keep the amount according to your decision. If you are a recipient, your earning is calculated as follows. Once all senders have made a decision, as many "h"-cards and "p"-cards will be put in an opaque box as there were "h"-choices and "p"-choices made by the senders. You will then draw one card from the box in private. If you draw an "h"-card, you will be paid £1.00. If you draw a "p"-card, you will be paid £3.50. The card will be put back into the box before the next recipient gets to draw a card. You will then be asked to fill in a questionnaire, will receive your earning and will leave the building. You will no longer be part of the experiment.

Five participants in this session have been randomly assigned to be recipients. The other participants have been assigned to be senders. The senders are in the experimental laboratory and the recipients are in the seminar room on the same floor. Thus, **you are a sender**. All participants are reading the same part-one instructions.

Please proceed on the screen and answer the control questions. You may use these instructions to answer the questions.

2

Figure S2: Part 1 instructions in the disclose condition (page 2)

Instructions (Part 2 and Part 3)

For the second part of the experiment you will be randomly assigned to be a "Person A" or a "Person B", and if you are assigned to be a Person A in the second part, you will be a Person B in the third part and the other way round. In both parts, once as a Person A and once as a Person B, you will be asked to make a decision in the following situation.

Person A will be endowed with £5, whereas Person B will receive no endowment. Person A will be asked to choose between action "n" and action "v" (see Figure 2, the letters "n", "v", "a" and "k" are arbitrary and have no meaning).

- If Person A chooses action "n", the £5 will be divided between Person A and Person B such that Person B will receive £2 and Person A gets to keep £3.
- If Person A chooses action "v", £6 will be added to the £5 and sent to Person B. Person B will be asked to choose between action "a" and action "k".
    - If Person B chooses action "a", the £11 will be divided between Person A and Person B such that Person A will receive £1 and Person B gets to keep £10.
    - If Person B chooses action "k", the £11 will be divided between Person A and Person B such that Person A will receive £5 and Person B gets to keep £6.

Figure 2

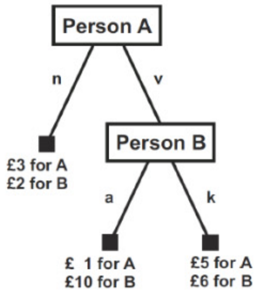

Before Person A makes a decision, Person B will have the opportunity to say how he or she decided in the first part of the experiment. Then, Person A will be asked to choose between action "n" and action "v" for each of the following cases:

- Person B said that his or her choice in the first part of the experiment was "h".
- Person B said that his or her choice in the first part of the experiment was "p".
- Person B did not say how he or she decided in the first part of the experiment.

At the end of the experiment, each Person A will be randomly paired with a Person B and your earnings will be calculated according to the above description and your and the other person's actual decisions. Note that Person B's decision (between "a" and "k") matters in determining his or her earnings only in the case that Person A chose action "v".

3

Figure S3: Part 2 and 3 instructions in the disclose condition (page 1)

CESS Instruction Sheet

Also note that the decision situation in the third part, when Person A and Person B roles are switched, is independent of the decision situation in the second part. That is, at the end of the experiment, you will be paired with a different person than for the second part and your earnings will be calculated according to your and the other person's actual decisions. However, only at the end of the experiment you will learn how the other persons decided in Part 2 and Part 3 and what your earnings are.

4

Figure S4: Part 2 and 3 instructions in the disclose condition (page 2)

S3. Screen shots

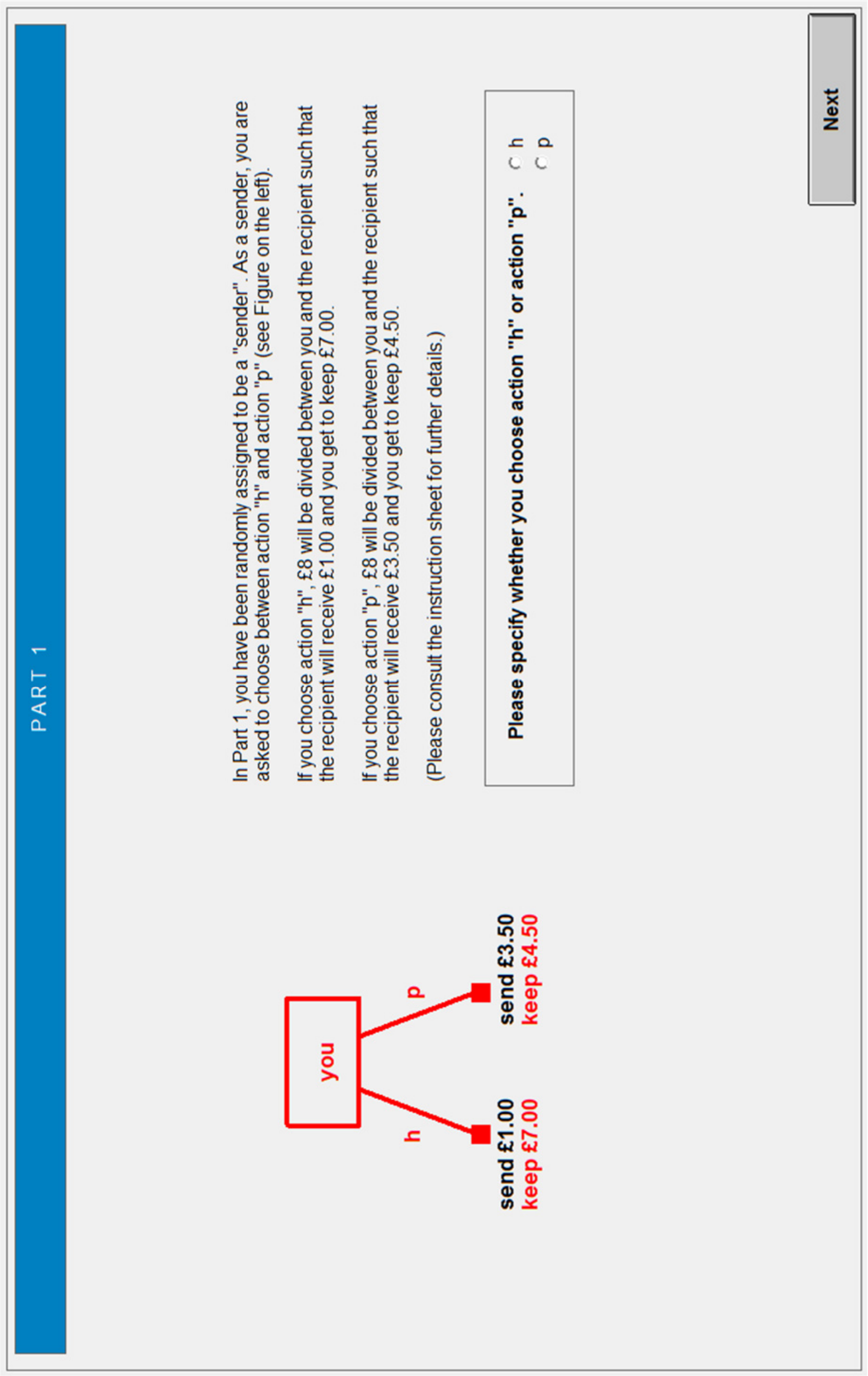

Figure S5: Screen shot of a sender's decision situation in the DG

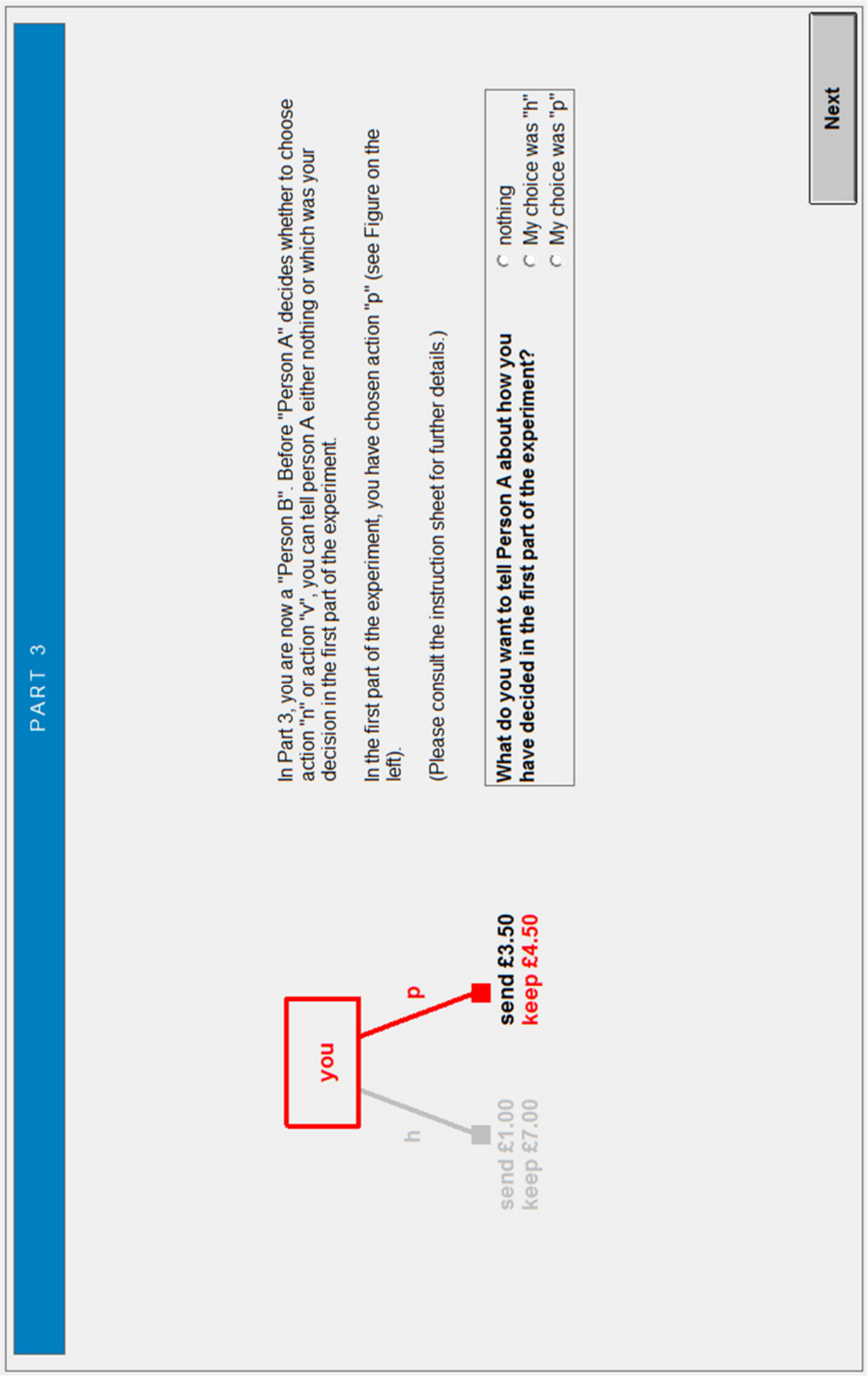

Figure S6: Screen shot of trustee's communication decision situation in the disclose condition

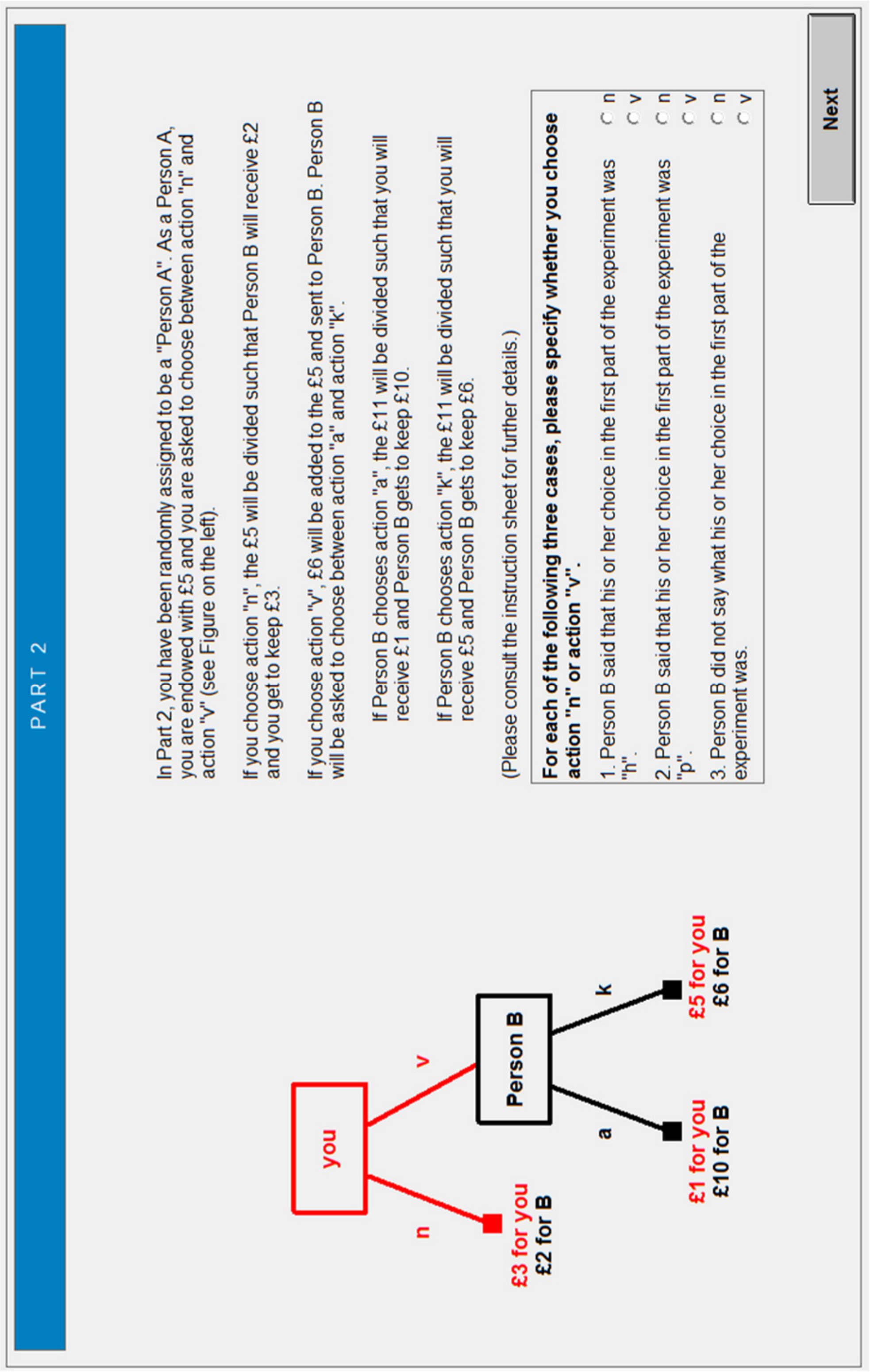

Figure S7: Screen shot of a truster's decision situation in the TG in the disclose condition

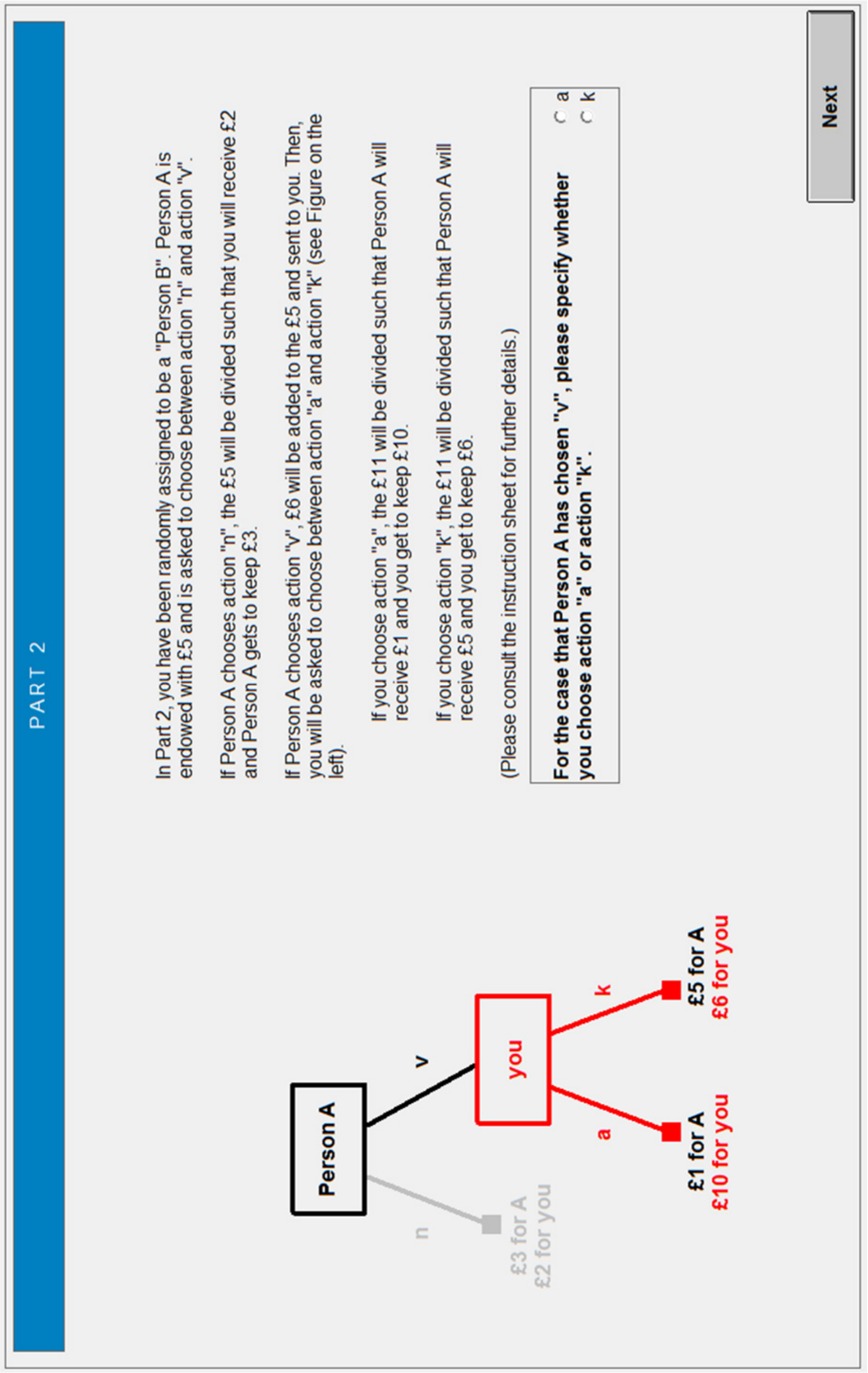

Figure S8: Screen shot of a trustee's decision situation in the TG

S4. Trustees' and trusters' behaviour and expected payoffs

Table S1: Observed frequencies and expected payoffs of trustees' decision sequences

	control (veiled) (n = 52)		disclose veiled (n = 53)		declare veiled (n = 53)		disclose unveiled (n = 57)	
decision sequence	freq. (in %)	exp. payoff (in £)	freq. (in %)	exp. payoff (in £)	freq. (in %)	exp. payoff (in £)	freq. (in %)	exp. payoff (in £)
gen.-return	17	8.34	-	-	-	-	-	-
gen.-nothing-return	-	-	0	7.78	6	8.1	0	7.34
gen.-mean-return	-	-	-	-	0	7.94	-	-
gen.-gen.-return	-	-	11	9.38	19	8.98	21	8.94
gen.-pocket	2	10.18	-	-	-	-	-	-
gen.-nothing-pocket	-	-	0	9.06	0	9.7	0	8.18
gen.-mean-pocket	-	-	-	-	0	9.38	-	-
gen.- gen.-pocket	-	-	2	12.26	4	11.46	11	11.38
mean-return	23	10.84	-	-	-	-	-	-
mean-nothing-return	-	-	15	10.28	6	10.6	16	9.84
mean-mean-return	-	-	8	10.36	9	10.44	2	9.64
mean- gen.-return	-	-	-	-	9	11.48	-	-
mean-pocket	58	12.68	-	-	-	-	-	-
mean-nothing-pocket	-	-	28	11.56	11	12.2	37	10.68
mean-mean-pocket	-	-	36	11.72	2	11.88	14	10.28
mean- gen.-pocket	-	-	-	-	34	13.96	-	-
exp. payoff (in £)		11.47		11.10		11.59		10.29

Notes: The table lists observed frequencies (in %) of trustees' decision sequences across experimental conditions and the corresponding expected payoffs (in £). The expected payoffs are calculated based on the actual frequencies of trusters' TG responses, which are displayed in Figure 3a. For example, a trustee in the declare veiled condition who chooses mean in the DG, lies and says to have chosen generous (gen.) and, given the opportunity, pockets in the TG (mean-gen.-pocket) earns £7 + 0.38 × £2 + 0.62 × £10 = £13.96 in expectation. The expected payoffs at the bottom of the table are the average expected payoffs per experimental condition.

Table S2: Observed frequencies and expected payoffs of trusters' strategies

	control (veiled) (n = 52)		disclose veiled (n = 53)		declare veiled (n = 53)		disclose unveiled (n = 57)	
strategy	freq. (in %)	exp. payoff (in £)	freq. (in %)	exp. payoff (in £)	freq. (in %)	exp. payoff (in £)	freq. (in %)	exp. payoff (in £)
send	46	2.6	-	-	-	-	-	-
send-send-send	-	-	23	2.36	25	2.96	14	2.57
send-send-keep	-	-	2	2.18	0	3.16	0	2.37
send-keep-send	-	-	8	2.92	13	2.82	7	2.81
send-keep-keep	-	-	0	2.74	2	3.02	0	2.61
keep	54	3	-	-	-	-	-	-
keep-send-send	-	-	8	2.62	6	2.94	0	2.99
keep-send-keep	-	-	2	2.44	6	3.14	2	2.79
keep-keep-send	-	-	34	3.18	19	2.80	40	3.23
keep-keep-keep	-	-	24	3.00	30	3.00	37	3.00
exp. payoff (in £)		2.82		2.88		2.96		3.01

Notes: The table lists observed frequencies (in %) of trusters' strategies across experimental conditions and the corresponding expected payoffs (in £). A truster's strategy defines his or her TG response to each possible bit of information about a trustee's DG behaviour. That is, for instance, by the strategy keep-keep-send a truster keeps if the trustee remains silent, keeps if the trustee reveals/says to have chosen mean and sends if the trustee reveals/says to have chosen generous in the DG. The expected payoffs are calculated based on the actual frequencies of trustees' TG responses, which are displayed in Table S1. For example, a truster who employs the strategy keep-keep-send in the disclose condition earns (0.15 + 0.28) × £3 + (0.08 + 0.36) × £3 + 0.11 × £5 + 0.02 × £1 = £3.18 in expectation. The expected payoffs at the bottom of the table are the average expected payoffs per experimental condition.

S5. Regression models

Table S3: Regression model estimations of trustees' communication choices and trusters' and trustees' TG choices

	M1 (revealed/said truth)		M2 (chose send in TG)		M3 (chose return in TG)	
	Coef	SE	Coef	SE	Coef	SE
DG choice in disclose veiled condition						
mean	1.674**	0.534				
generous	+∞	-				
DG choice in declare veiled condition						
mean	(ref.)					
generous	3.060***	0.784				
DG choice in disclose unveiled condition						
mean	0.470	0.585				
generous	+∞	-				
control condition			(ref.)			
stated DG choice in treatment conditions						
mean or nothing			-0.720*	0.318		
generous			0.775*	0.324		
DG choice in veiled conditions						
mean					(ref.)	
generous					2.824***	0.569
DG choice in unveiled condition						
mean					-0.187	0.416
generous					1.571**	0.537
const.	-1.674***	0.445	-0.154	0.279	-0.878***	0.196
N_1	138		541		215	
N_2			215			
pseudo R^2	0.15		0.08		0.16	
$\chi^2_{(df)}$	26.64***		74.02***		46.20***	

Notes: The table lists coefficient estimates from logistic regressions (*** $p < 0.001$, ** $p < 0.01$, * $p < 0.05$, for two-sided tests). Results shown in Figures 2a and 2b in the main part of the article are based on model estimations M1 and M3, respectively. In model M1 two coefficients could not be estimated because all subjects chose to reveal their DG choice in these conditions. Model M2 is estimated with cluster-robust standard errors. N_1 denotes the number of decisions and N_2 denotes the number of clusters.

Table S4: Regression model estimations of trusters' TG choices

	M1 (chose send in TG)		M2 (chose send in TG)		M3 (chose send in TG)		M4 (chose send in TG)	
	Coef	SE	Coef	SE	Coef	SE	Coef	SE
control condition	(ref.)		(ref.)		(ref.)		(ref.)	
revealed DG choice in disclose veiled condition								
nothing	-0.596	0.406	-0.585	0.437	0.211	0.610	-1.792*	0.786
mean	-0.511	0.403	-0.482	0.418	0.041	0.623	-1.340	0.794
generous	1.084**	0.414	1.387**	0.426	1.953***	0.576	0.288	0.981
stated DG choice in declare veiled condition								
nothing	-0.267	0.396	-0.510	0.441	0.562	0.622	-1.792*	0.741
mean	-0.428	0.400	-0.704	0.434	0.174	0.651	-1.792*	0.741
generous	0.655	0.398	0.617	0.408	1.052	0.602	-0.087	0.832
revealed DG choice in disclose unveiled condition								
nothing	-1.168**	0.429	-1.358**	0.437	-1.376	0.862	-1.974**	0.761
mean	-1.520***	0.459	-1.758***	0.494	-0.940	0.759	-2.773***	0.791
generous	0.618	0.390	0.780*	0.388	1.022	0.573	1.253	1.205
actual DG choice								
generous = 1			-0.017	0.338				
actual TG choice as trustee								
return = 1			1.858***	0.314				
const.	-0.154	0.279	-0.910**	0.292	-1.427**	0.456	1.792**	0.627
N_1	541		541		322		219	
N_2	215		215		128		87	
pseudo R^2	0.10		0.21		0.12		0.17	
$\chi^2_{(df)}$	74.08***		95.39***		39.06***		46.86***	

Notes: The table lists coefficient estimates with cluster-robust standard errors from logistic regressions (*** $p < 0.001$, ** $p < 0.01$, * $p < 0.05$, for two-sided tests). In all models, the subject's TG choice as truster (send = 1) is the binary dependent variable. Results shown in Figures 3a, 3b and 3c in the main part of the article are based on model estimations M1, M3 and M4, respectively. Models M3 and M4 are estimated with cases where subjects chose pocket or return as trustees in the TG, respectively. N_1 denotes the number of decisions and N_2 denotes the number of clusters.

References

S1. Fehr E, Schmidt KM (1999) A Theory of Fairness, Competition, and Cooperation. Q J Econ 114: 817-868.

S2. Blanco M, Engelmann D, Normann HT (2011) A within-subject analysis of other-regarding preferences. Game Econ Behav 72: 321-338.

S3. Binmore K, Shaked A (2010a) Experimental economics: Where next? J Econ Behav Organ 73: 87-100.

S4. Binmore K, Shaked A (2010b) Experimental Economics: Where Next? Rejoinder. J Econ Behav Organ 73: 120-121.

S5. Fehr E, Schmidt KM (2010) On inequity aversion: A reply to Binmore and Shaked. J Econ Behav Organ 73: 101-108.

S6. Rabin M (1993) Incorporating Fairness into Game Theory and Economics. Am Econ Rev 83: 1281-1302.

S7. Bolton GE, Ockenfels A (2000) ERC: A Theory of Equity, Reciprocity, and Competition. Am Econ Rev 90: 166-193.

S8. Falk A, Fischbacher U (2006) A theory of reciprocity. Game Econ Behav 54: 293-315.

S9. Crawford VP, Costa-Gomes MA, Iriberri N (2013) Structural Models of Nonequilibrium Strategic Thinking: Theory, Evidence, and Applications. J Econ Lit 51: 5-62.
